# Colorado integrated behavioral health plus (CIBH+): aligning behavioral health within a generalist approach to primary care

**DOI:** 10.3389/fpsyg.2025.1523369

**Published:** 2025-04-23

**Authors:** Jay H. Shore, Maryann Waugh, Shandra Brown Levey, Jacqueline Calderone, Corey Lyon, Jodi Summers Holtrop, R. Mark Gritz, Vanessa Owen, Shannon K. McWilliams, Frank deGruy

**Affiliations:** ^1^Department of Psychiatry, University of Colorado Anschutz Medical Campus, Aurora, CO, United States; ^2^Department of Family Medicine, School of Medicine, University of Colorado Anschutz Medical Campus, Aurora, CO, United States; ^3^Department of Medicine, School of Medicine, University of Colorado Anschutz Medical Campus, Aurora, CO, United States

**Keywords:** integrated care approach, psychology, multidiscipliary team, primary care, comprehensive healthcare, mental health, generalism, general practice

## Abstract

As healthcare costs and physician burnout in the U.S. escalate, and the acuity and prevalence of behavioral health issues hit historical highs, it is critically important that we continue to evolve care approaches that can deliver good health and well-being at the population level. Colorado Integrated Behavioral Health Plus (CIBH+) uses a whole-person health perspective, aligning psychologists, primary care physicians, and other specialists, within a generalist approach to primary care. Here, we document our local experience in services delivery, including the rationale for the CIBH+ approach, key implementation elements, and the ability to mitigate population, patient, and provider challenge by building upon existing clinically-and cost-effective models of integrated care. With this description, we hope to spark optimism, enthusiasm, and ongoing innovation in other multidisciplinary care teams seeking ways to improve patient and provider experience.

## Introduction

Colorado Integrated Behavioral Health Plus (CIBH+) uses a whole-person health perspective to deliver comprehensive integrated care in the primary care setting, building upon existing clinically-and cost-effective models of integrated care. Here, we describe the rationale for the CIBH+ approach, detailing key implementation elements and its ability to mitigate population, patient, and provider challenges. With this description, we hope to spark optimism, enthusiasm, and ongoing innovation in other behavioral health and primary care providers seeking new ways to improve patient and provider experience.

### The importance of a population health approach

There is a growing realization that the U.S. healthcare system will need to shift to a population health approach if we are to stem the tide of escalating healthcare costs, poor health outcomes, and patient disengagement. In the U.S., healthcare outcomes lag behind countries with systems designed to support population health; while U.S. healthcare costs continue to top international rankings (Howard, 2023; Schäfer et al., 2019). Fundamental to a population-based approach is assuming responsibility for the health of the larger population, not just the patients who are able to access and engage with the healthcare system (Tepper et al., 2022). Behavioral health, encompassing mental health and substance use disorders, is a particularly critical population-level concern in the U.S. One in eight people live with a mental disorder (World Health Organization, 2022.), and 8% of emergency room visits included a behavioral health condition as the principal diagnosis. Rates are even higher (13%) for the Medicaid population, and the large majority of those in need are unable to access effective behavioral health care (National Institute of Mental Health, 2024; Stoddard et al., 2023; World Health Organization, 2022). Meanwhile, provider shortages, particularly in behavioral health, exacerbate patient access challenges and provider burnout (National Centers for Health Workforce Analysis, 2023).

### The role of primary care in population health

At the heart of population health is comprehensive primary care which is positively associated with population level health outcomes, quality, equity, and lowered cost (Stange et al., 2023). A contemporary understanding of primary care acknowledges the generalist approach as fundamental to its efficacy. The generalist approach, well described by Reeve et al. (2011), focuses on the person not disease, and is continuous, not episodic. The generalist approach leverages adaptable, iterative processes to provide whole person care. Care is informed by primary care physicians’ (PCPs’) broad knowledge, and it is guided by a personal relationship between PCPs and patients— doctors know their patients by name—honoring each patient’s values, family, and social context. Within this approach PCPs are adjustable, continually reprioritizing care to remain aligned with each patient’s changing life stage, goals, and health status (Reeve et al., 2011; Stange, 2009). Most people receive behavioral treatment outside of traditional behavioral health settings (Van Beek et al., 2008), making behavioral health a component of most primary care visits (Schrager, 2021) and a critical component of the generalist approach. In the U.S., anxiety, depression, and substance use disorders are the most prevalent behavioral health disorders, and those most frequently encountered in the primary care setting. Annual prevalence data shows that 19% of adults are diagnosed with anxiety, 8% with major depression, 4% with post-traumatic stress disorder, 3% with bipolar disorder, and 1% or less with each of borderline personality disorder, obsessive compulsive disorder, and schizophrenia. In 2021, 35% of those with mental illness were diagnosed with a co-occurring substance use disorder (National Alliance on Mental Illness, 2023).

## Context

CIBH+ attempts to solve the problem of population-level behavioral-health need and the artificial separation of behavioral and physical health. It was developed to provide whole-person care within a flexible generalist approach to primary care, and it differs from other integrated care models in a few significant ways. These include more flexible care team roles and expanded responsibilities for behavioral health providers (see Tables 1, 2 for details). The current CIBH+ implementation builds on more than 20 years of iteration, driven by ongoing adaptation to local needs and priorities. In the early 2000s, behavioral health and family medicine practitioners at the University of Colorado (UC) began meeting to develop new care approaches, recognizing the ubiquity of behavioral health problems in primary care (Donaldson et al., 1996) and the need to increase patient access to high quality behavioral healthcare. Noting the growing evidence base of collaborative care models (CoCM), which were focused only on depression at that time, we began using part-time psychologists to provide depression care in the primary care setting. Over time, however, the embedded psychologist team migrated to the core of the CIBH+ model, moving from a disease-specific to a person-centered comprehensive behavioral health approach; organically shifting away from the more prescriptive programmatic CoCM model to an approach that optimizes the pragmatic and broad training of psychologists who serve an expanded set of patients. CIBH+ psychologists assume a more extensive personal care role compared to traditional CoCM. They initiate brief behavioral health care targeted at problems that may or may not be diagnostically driven, offering both immediate support as well as conventional primary care medical interventions.

**Table 1:** Core components of CIBH+.

CIBH+ component	Description
A care team with a flattened hierarchy	Traditional PCPs, embedded psychologists, and embedded/remote psychiatrists. No role or person is more important than another.
Triage	Universal screening and specialty evaluations including addiction medicine and child psychiatry.
Stepped care	Includes population-based screening, evidence-based interventions, inter-disciplinary care, and intensive interventions for acute care when needed.
Patient-centered	Care plans based on patient’s values, preferences, and treatment goals.
Psychiatry	Psychiatric evaluations for short-term care, up to six direct-care patient therapy sessions, and home visits as needed.
Psychology	Behavioral health evaluations and testing, direct-care patient therapy sessions, and home and hospital visits as needed.
Team culture	Focused on flexibility, humility, and shared responsibility.
Digital tools	Use of technology to drive efficiency and team-based care.
Intentional use of a/synchronous communication	Care team leverages in-person and virtual provider-to-provider consultation and tele-psych huddles with behavioral health staff across multiple clinics.
Supported referral	Helping patients with the most complex or chronic behavioral health conditions access long-term specialty care as needed, while reducing demand by supporting many patients within primary care. Leverage existing relationships with community mental health centers and the Regional Accountable Entities for Medicaid and indigent patients, and Colorado’s independent providers for Medicaid, commercially insured, and self-pay patients.

**Table 2:** Collaborative care model components and CIBH+ modifications.

CoCM component (Advancing Integrated Mental Health Solutions (AIMS) Center, 2023)	CIBH+ modification
Treats a specific and predetermined population with a specified set of conditions	Treats all conditions and all patients. For acute/complex cases, refer to specialized behavioral healthcare while remaining an informed and engaged medical home.
Programmatic orientation: patients are identified, recruited for CoCM program participation, and return to treatment as usual upon program completion.	Individualized care as the norm: triage results and patient health goals drive care plan and care team composition.
Psychiatrists provide consultation but no direct patient care.	Psychiatrists support PCP and BHP with consultation but also provide direct patient care as needed.
PCP leads the care team.	Flattened care team hierarchy; individual care plans are led by the provider (PCP, BHP, care coordinator) most appropriate for the patient. Psychologists often lead care teams for patients with behavioral health needs.
Psychologists play a contained role on the care team.	Psychologists play an expanded role on the care team, initiating behavioral health treatment, engaging psychiatric and other providers as needed, and leading coordination and cross-disciplinary education across the care team.
Care decisions, including CoCM treatment completion, are based on measurement-based care targets.	Stepped care decisions incorporate measurement-based care goals as well as patient perspective on goal progression and treatment success.

This shift expanded behavioral health access to more patients, but it still left primary care teams unable to support the needs of a significant proportion of their patient panel, particularly with complex situations such as multiple diagnoses, complicated medication need, and acute symptoms. In 2011, psychiatrists joined the primary care integration team to treat patients whose health issues required deeper psychiatric expertise and prescribing (hence, the “plus” in CIBH+). This was particularly advantageous for patients with extensive comorbidities, failure in first line treatments, and patients who needed specialty behavioral health guidance to better understand their mental health conditions to adhere to treatment. Each psychiatrist supported multiple primary care clinics with part-time, onsite availability based on need at each clinic.

In 2014, informal needs assessment with the integrated care providers revealed 1. A need for psychiatric practitioners to fulfill a dual-role – both to continue to provide direct care to the most complex/acute/treatment-resistant patients and to have dedicated time to consult with PCPs, collaboratively improving PCPs knowledge and ability to medically manage behavioral health conditions. 2. A need to expand physical space in the clinics for direct psychiatric patient care and private provider collaboration. Our ability to meet these needs was accelerated by two timely breakthroughs. First was the addition of telehealth; the value of which cannot be overstated. While virtual care was a solution originally designed to mitigate physical space limitations, it quickly amplified the impact of the approach by allowing a psychiatrist to become embedded within a primary care clinic without spending time on travel. A second breakthrough was the recognition by Colorado funders that to implement comprehensive, whole-person care, and to improve the ability of a primary care clinic to render care to a greater swath of its patient panel, PCPs and psychologists had to improve their own ability to support complex and acute behavioral health conditions, and specialists would need to evolve their ability to flexibly support care within the primary care system. Providers needed time to learn about each other’s care processes and identify the diagnostic and medication support needed to best meet patient and PCP needs in real time. Genuine collaboration across these team members was identified as critical to coordinate care across multiple health issues and develop effective care plans.

This historical context explains how addressing behavioral health issues became key to effective primary care and how this approach evolved within a system focused on comprehensive care. The CIBH+ approach can best be conceptualized not as competition to other collaborative models, but an extension and modification that optimized integrated care delivery within this philosophical orientation to primary care, and within clinics of diverse sizes and resources. With more comprehensive and generalized notions of primary health care re-emerging globally (Bitton, 2018), this local solution has relevance and interest a growing number of providers and systems.

## Innovation details

### The core components of CIBH+

The core components of CIBH+ (Table 1) improve provider experience using team-based care to enable top of scope work, create a positive and collaborative work culture, and improve perceived efficacy, which in traditionally siloed health settings, is increasingly challenged by patients’ complex co-morbid behavioral and physical health needs.

CIBH+ is integrated care delivered within a generalist approach to primary care. In CIBH+, psychologists are embedded into primary care practices developing relationships with clinical and administrative staff members and helping to define clear understandings of all staff members’ roles within the clinic. Primary care psychologists are trained with a generalist approach. They support a population of patients of all ages, assess individual patient strengths, needs, and values, conduct psychological evaluations and testing, manage crisis situations, organize multiple supports, and guide care accordingly. Dovetailing with primary care processes, CIBH+ psychologists direct care plans for patients with psychological and physical health diagnoses and engage patients in care via stepped care approach driven by individual patient context rather than diagnoses. Team roles are flexible, and all primary care team members support behavioral care blending in-person communication and digital tools including videoconferencing.

Psychiatrists specialize in complex and acute behavioral health disorders, providing assessment, diagnosis, and support through medication and behavioral recommendations. While psychiatrists and psychologists expand the behavioral health capacity of primary care teams, psychologists along with care managers support coordination with specialty care and social support for patients with complex cross-systems involvement and social needs. While the psychologist, the PCP, and/or the care manager maintain(s) the personal connection to each patient depending on care needs, case conceptualization prioritizes broad and wide-ranging input, and team-based care plans prioritize individualized patient goals, and family and cultural considerations. Medical assistants and other support staff support providers working at the top of their scope, optimizing a provider’s clinical care.

The following example, taken from composites, illustrates how CIBH+ works. Based on an elevated depression screening score and concerns identified by the PCP, the embedded onsite psychologist identifies a patient, Marco, with paranoia, chronic hypertension, and a variety of psychosocial concerns including low income. As part of holistic triage, the psychologist assesses existing clinical history, noting high symptom acuity and suspicions of schizoaffective disorder. Based on this assessment, the psychologist arranges a psychiatric assessment for diagnostic clarity. The psychologist facilitates the varied and ongoing communication needed to engage the members of the care team (PCP, psychologist, psychiatrist, and care manager), the patient, as well as important others (in Marco’s case, a concerned daughter) to integrate care. An administrative team member helps to schedule Marco for a virtual psychiatric assessment and facilitates a virtual provider-to-provider consultation for the PCP and psychiatrist to discuss the assessment results within the context of Marco’s multiple diagnoses and symptoms. Using a whole-person biopsychosocial wellness conceptualization, the psychologist collects team input, coordinates treatment priorities, and develops a care plan that is aligned with Marco’s current life goals and cultural orientation. He is initially focused on addressing the paranoia, with little interest medication, nor in exercise or food-related changes that could improve his hypertension.

Over time, Marco builds a stronger relationship with his PCP and participates in direct care therapy sessions with the psychologist. The team helps Marco enroll in the local Medicaid Program, alleviating the stress of prescription costs. Marco becomes more adept at managing his behavioral health symptoms, and when he finds out that he is going to be a grandfather, he becomes motivated to address his hypertension. Marco maintains an ongoing and personal relationship with his CIBH+ care team members who evolve his care plan with his changing goals, shifting focus to health behaviors related to diet and exercise, and providing him with education about medication that could help with the hypertension. An administrative team member ensures that Marco’s health records remain updated with key information documented.

**Figure 1:**
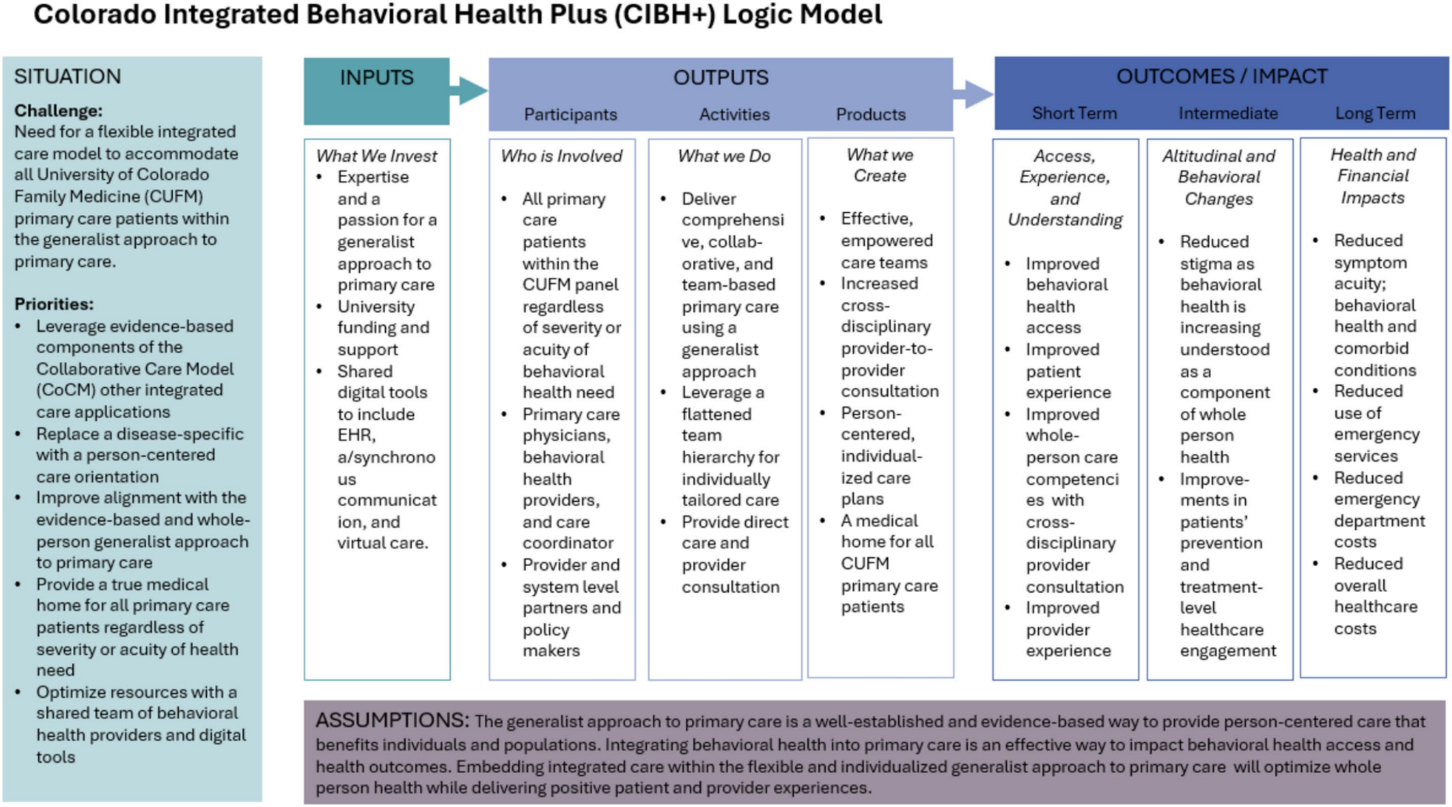
CIBH+ logic model.

In CIBH+, team-based care (outlined in the example above), is accomplished with an interdisciplinary approach looking at a broad array of factors, such as medication adherence, health behaviors, social determinants, and cultural context. The psychologist works to provide immediate support, initiating targeted episodic behavioral health interventions and traditional primary care medical interventions. Psychiatric care team members most frequently support medication management, working with the onsite psychologist to make sure all team members receive relevant psychoeducation. The psychologist ensures that everyone understands how each care component, from therapy, to medication, to social supports and referral, contributes to a comprehensive care plan. For the smaller subset of patients whose issues are driven by a severe behavioral health disorder, a referral to long-term specialized care is utilized. In such cases, the integrated primary care team provides gap services, helping to mitigate long wait times for specialty care. Please see Figure 1, CIHB+ logic model.

### Comparing CIBH+ to CoCM

As noted previously, CIBH+ extends and adapts traditional collaborative care models to optimize integrated care within the generalist approach to primary care. The five core principles of CoCM as outlined by the AIMS center, (1) patient-centered care team, (2) population-based care, (3) measurement-based care, (4) evidence-based care, and (5) accountable care payment components (Advancing Integrated Mental Health Solutions (AIMS) Center, 2023) are all present in the CIBH+ approach as well. Key differences are few, but significant. These are outlined in Table 2.

### CIBH+ today

CIBH+ has been implemented in all seven family medicine clinics since 2020 and has been active in its current state in some clinics since as early as 2014. All clinics are located in metro Denver, Colorado and range in size from four to 54 full-time equivalent providers. The cross-clinic team of behavioral health providers includes, on average, six full-time psychologists, 12 part-time psychologists, and10 part time psychology interns/externs, as well as 11 part-time psychiatrists and one part-time psychiatry resident. On average, the CIBH+ program is supported by 3.1 FTE of psychiatry and 15.1 FTE of psychology per month. Each of the seven CIBH+ clinics has an onsite medical care manager/social worker. Clinics ranged in size with the largest clinic accounting for 110,807 (27.9%) of total encounters, and the smallest clinic accounting for 21,351 (5.4%) of total encounters. Please see Table 3 for descriptive patient detail.

**Table 3:** Description of patient participants in CIBH+.

	Total patient population (*N* = 74,177)*
Gender identification	
Female	27,683 (37%)
Male	20,491 (28%)
Non-binary	414 (1%)
Prefer to self-describe/Prefer not to answer/missing	25,589 (34%)
Age (Median)	42
Race/Ethnicity	
American Indian or alaska native	489 (1%)
Asian	4,429 (6%)
Black or african-american	4,512 (6%)
Hispanic	7,871 (11%)
Native Hawaiian or Pacific Islander	197 (<1%)
White or Caucasian	52,548 (71%)
Mixed or multi-racial/race not specified	2,134 (3%)
Prefer not to answer/missing	2,856 (4%)
Payor status	
Medicaid	8,827 (12%)
Medicare	12,907 (17%)
Encounters	
Total encounters	397,124
Encounters per patient (Median)	4
Total behavioral health encounters	26,425
Psychology encounters	23,034
Psychiatry encounters	3,391
Behavioral health encounters per patient with >0 behavioral health encounters (Median)	7

^*^ Based on EHR visit data from all participating practices from July 1, 2020 – June 30, 2023, de-duplicated at the patient level by unique patient ID.

The 26,425 encounters with CIBH+ providers account for 6.7% of the total clinic encounters, but wholly underrepresent the full extent of whole-person behavioral health care. As part of the CIBH+ program, behavioral health providers also consult with primary care providers, providing case-specific consultation and education that, by design, improve the capacity of primary care providers to address behavioral health issues over time. In a random sample of 1,083 encounters with PCPs, 198 (18%) were with patients with at least one behavioral health condition noted as part of the encounter. Mixed methods analysis with a smaller subsample of patients shows that patients described their overall experiences with CIBH + as positive and appreciated having behavioral healthcare as part of primary care. CIBH + effectively improved participant’s perceived access to and quality of behavioral health care (Gurfinkel et al., 2024). An in-depth analysis of patient clinical profiles and outcomes is the focus of forthcoming work.

## Discussion

### CIBH+: a generalist primary care approach

The value of a generalist, primary care approach has been described as exceeding the sum of its parts; representing a personalized care experience not captured with individual clinical process or outcome metrics (Etz et al., 2019). The nationally recognized, Person-Centered Primary Care Measure (PCPCM) is an 11-item patient-report tool with strong psychometric properties. It measures 11 components of high-value primary care according to patients, clinicians, and payers. CIBH+ is well-aligned with the overall construct of the PCPCM (high-value primary care) and is particularly well suited to improve six of the 11 care components: (1) accessibility, (2) a comprehensive, whole-person focus, (3) integrating care across acute and chronic illness, prevention, mental health, and life events, (4) coordinating care in a fragmented system, (5) goal-oriented care, and (6) disease, illness, and prevention management. Perhaps even more importantly, as described by Stange et al. (2023) an effective generalist approach applies these 11 components using the following three rules, implemented iteratively:

1. Recognize a broad range of problems/opportunities/capacities.
2. Prioritize action with the intent to promote health, healing, and connection.
3. Personalize care based on the particulars of the individual or family in their local context (Stange et al., 2023).

These rules are not just well-aligned with, but are explicitly embedded within, the CIBH+ approach. Recognizing a broad range of problems and opportunities is part of the foundational training of both the PCP and the psychologist. With formal didactic training and provider-to-provider consultation, as well as formal and informal teamwork, the specialized training of the psychiatrist amplifies recognition of behavioral health problems and treatment opportunities across the team. This significantly enhances comprehensive case conceptualization within a whole-person vision for all members of the care team – including the patient. This, in turn, enables more accurate prioritization and action for health and healing. The integration of the primary care team (physically and virtually) also accelerates opportunities for action, mitigating the high no-show risk of a traditional behavioral health referral and focusing a team with diverse skill sets on shared care priorities. For example, in the anecdote above, Marco was able to receive immediate Medicaid enrollment support because his CIBH+ care included assessment and triage that revealed financial as well as health concerns, and the enrollment expertise of the care manager. Patients engaged in traditional primary and/or behavioral health care would not likely encounter a team ready to address such entangled social and health issues.

Finally, if most primary care visits include a behavioral component, then to be personalized and effective, primary care plans must also address behavioral health. The flattened hierarchy of the CIBH+ care team and the culture built intentionally to encourage flexibility, humility, and shared responsibility, focuses team members’ attention on the individual needs and priorities of the patient in each moment, not on just one provider’s expertise, a specific diagnoses, or organizational structure.

### Integrated care: from specialist to generalist approach

There are evidence-based integrated care models that very effectively bring behavioral health into the primary care environment. These models are designed to deliver care and improve cost and outcomes for patients with specific diseases and comorbidities, such as diabetes and depression. The clinical advances and research related to such programs have made invaluable contributions to an understanding of integrated care and have advanced payment opportunities. In Colorado, we too began with a disease-specific model. We shifted to a comprehensive care approach over time, recognizing that the presence or lack of a diagnosis should not limit access to whole person care, including behavioral health care. Marco is a good example of a patient needing more than a condition-specific intervention, benefiting significantly from comprehensive and coordinated care. CIBH+ exemplifies an approach in which providers leverage condition-specific expertise, within a generalist, team-based primary care approach to address entangled physical, behavioral, and social challenges.

In Colorado, we have implemented CIBH+ across seven different clinics of one large family medicine system. Our approach is designed for clinic-level variability in service delivery and team-based care configurations to align with each clinic’s patient population, staffing resources, and physical layout. A shared team of psychologists and psychiatrists and digitally supported synchronous and asynchronous communication empowers collaboration across the seven clinics, adapting to diverse primary care settings. In other countries, stronger primary care support is associated with better population level outcomes and overall healthcare expenditures (Shi, 2012; Stange et al., 2023), and team-based care is also associated with improved patient and provider experiences (Hopkins and Sinsky, 2022; Lyon et al., 2018). There are no anticipated near-term solutions for provider shortages (National Centers for Health Workforce Analysis, 2023), and as noted by a recent National Academies of Medicine panel, sustainable approaches to care must move away from an uncoordinated approach where multiple individual providers are all responsible for pieces of the same individuals, and optimize the strength and camaraderie of care teams to manage populations (National Academies, 2024).

In implementing and evolving this approach over several decades, we have learned many key lessons, and this team-based mindset is chief among them. Early challenges included learning how to intentionally cultivate a culture of collaboration and humility and celebrate team ‘wins’ over individual expertise. This is critical to the success of team-based care. Providers who cannot work within a flattened hierarchy, where the input of a care manager, medical assistant, or the patient herself are valued equally to that of physicians and behavioral health professionals, will not be successful in an approach like CIBH+. Interprofessional relationship building is foundational to a collaborative culture, and we have found how important it is for each clinic to establish individualized opportunities and schedules for team meetings, discussions, daily huddles, and impromptu provider conversations. While this is a best practice we identified in our work locally by taking seriously the feedback of clinical team participants, it is also a concept echoed in global conversations about integrated care best practices (Kearney et al., 2020) and cannot be understated. It is also critical to have supportive and adaptive leaders, those whose goal is to optimize success and positive experiences for patients and providers by empowering participants at all levels to engage in decision making and effective change management.

### Contributing to improved population health

Assuming care for a larger population, including individuals who are not able, or do not access or engage in care, is a system-level effort that encompasses payers and community organizations complementing the efforts of providers (Health Care Transformation Task Force and the National Association of ACOs, 2024; Jager, 2023; Struijs et al., 2015). As system-level entities like payers, and accountable care organizations accept and forward critical population health initiatives like improving health literacy and outreaching non-engaged members, the greater the impact of provider-level efforts like integrated care. In Colorado, for example, 99% of Medicaid beneficiaries are enrolled with accountable care organizations working to improve members’ behavioral and physical health engagement, using a population health approach that centers on expanding the role of primary care (Health Management Associates, 2024). Innovations like CIBH+ play a significant role in system-level efforts to effectively expand the role of primary care. By recognizing the critical behavioral health component to whole-person health, leveraging the existing and natural alignment of psychology and the generalist approach to primary care, improving the ability of primary care to serve an expanded set of behavioral health needs with psychiatry, and leveraging effective team-based care to coordinate across complex needs, there is a significant potential to improve patient and provider experiences and drive improvements in healthcare costs and outcomes.

## Data Availability

The original contributions presented in the study are included in the article/supplementary material, further inquiries can be directed to the corresponding authors.

## References

[ref1] Advancing Integrated Mental Health Solutions (AIMS) Center, (2023). Principles of collaborative care. Available online at: https://aims.uw.edu/principles-of-collaborative-care/ (Accessed March 5, 2025).

[ref2] Bitton A. (2018). The necessary return of comprehensive primary health care. Health Serv. Res. 53, 2020–2026. doi: 10.1111/1475-6773.12817, PMID: 29285762 PMC6051987

[ref3] Donaldson M. S. Yordy K. D. Lohr K. N. Vanselow N. A. (1996). “Defining primary care” in Primary care: America’s health in a new era. eds. M. S. Donaldson, K. D. Yordy, K. N. Lohr, N. A. Vanselow (Washington, D.C., National Academies Press (US)). 25121221

[ref4] Etz R. S. Zyzanski S. J. Gonzalez M. M. Reves S. R. O’Neal J. P. Stange K. C. (2019). A new comprehensive measure of high-value aspects of primary care. Ann. Fam. Med. 17, 221–230. doi: 10.1370/afm.2393, PMID: 31085526 PMC6827628

[ref5] Gurfinkel D. Owen V. Kreisel C. Hosokawa P. Kluger S. Legge C. . (2024). Patient perspectives of integrated behavioral health in primary care: a mixed methods analysis. J. Patient Exp. 11:23743735241293877. doi: 10.1177/23743735241293877, PMID: 39497928 PMC11533315

[ref6] Health Care Transformation Task Force and the National Association of ACOs, (2024). Reimagining beneficiary engagement in accountable care models. Available online at: https://hcttf.org/wp-content/uploads/2024/07/Reimagining-Beneficiary-Engagement-in-Accountable-Care-Models_Final-1.pdf (Accessed March 18, 2025).

[ref7] Health Management Associates, (2024). Who’s on second? What’s a RAE and BHASO and why do they matter for Colorado behavioral health services? Available online at: https://hcpf.colorado.gov/sites/hcpf/files/Whos%20on%20Second.pdf (Accessed March 18, 2025).

[ref8] Hopkins K. D. Sinsky C. (2022). Taking team-based care to the next level. Fam. Pract. Manag. 29, 25–31. 35536298

[ref9] Howard J., (2023). U.S. spends most on health care but has worst health outcomes among high-income countries, new report finds. CNN. Available online at: https://www.cnn.com/2023/01/31/health/us-health-care-spending-global-perspective/index.html (Accessed December 16, 2023).

[ref10] Jager A. J. (2023). Reimagining “covered lives” as communities: communitarian ethics for ACOs. Am. J. Accountable Care 11, 32–38. doi: 10.37765/ajac.2023.89383

[ref11] Kearney L. K. Zeiss A. M. McCabe M. A. Thistlethwaite J. E. Chana N. Chen S. . (2020). Global approaches to integrated care: best practices and ongoing innovation. Am. Psychol. 75, 668–682. doi: 10.1037/amp0000490, PMID: 31393143

[ref12] Lyon C. English A. F. Smith P. C. (2018). A team-based care model that improves job satisfaction. Fam. Pract. Manag. 25, 6–11, PMID: 29537246

[ref13] National Academies, (2024). Addressing workforce challenges across the behavioral health continuum of care: A workshop. Available online at: https://www.nationalacademies.org/event/41726_07-2024_addressing-workforce-challenges-across-the-behavioral-health-continuum-of-care-a-workshop (Accessed October 5, 2024). 39993048

[ref14] National Alliance on Mental Illness, (2023). Mental health by the numbers. Available online at https://www.nami.org/about-mental-illness/mental-health-by-the-numbers/ (Accessed March 21, 2025).

[ref15] National Centers for Health Workforce Analysis, (2023). Behavioral health workforce 2023 (research brief). Health Resources and Services Administration. Available online at: https://bhw.hrsa.gov/sites/default/files/bureau-health-workforce/Behavioral-Health-Workforce-Brief-2023.pdf (Accessed November 5, 2024).

[ref16] National Institute of Mental Health, (2024). Mental health information statistics. Available online at: https://www.nimh.nih.gov/health/statistics/mental-illness (Accessed October 4, 2024).

[ref17] Reeve J. Irving G. Dowrick C. F. (2011). Can generalism help revive the primary healthcare vision? J. R. Soc. Med. 104, 395–400. doi: 10.1258/jrsm.2011.110097, PMID: 21969476 PMC3184534

[ref18] Schäfer W. L. A. Boerma W. G. W. van den Berg M. J. De Maeseneer J. De Rosis S. Detollenaere J. . (2019). Are people’s health care needs better met when primary care is strong? A synthesis of the results of the QUALICOPC study in 34 countries. Prim. Health Care Res. Dev. 20:e104. doi: 10.1017/S1463423619000434, PMID: 32800009 PMC6609545

[ref19] Schrager S. (2021). Integrating behavioral health into primary care. Fam. Pract. Manag. 28, 3–4, PMID: 33973750

[ref20] Shi L. (2012). The impact of primary care: a focused review. Scientifica (Cairo) 2012:432892. doi: 10.6064/2012/432892, PMID: 24278694 PMC3820521

[ref21] Stange K. C. (2009). The generalist approach. Ann. Fam. Med. 7, 198–203. doi: 10.1370/afm.1003, PMID: 19433836 PMC2682975

[ref22] Stange K. C. Miller W. L. Etz R. S. (2023). The role of primary care in improving population health. Milbank Q. 101, 795–840. doi: 10.1111/1468-0009.12638, PMID: 37096603 PMC10126984

[ref23] Stoddard D. Darby B. Gray T. Spear C. (2023). Access across America: State-by-state insights into the accessibility of care for mental health and substance use disorders (Milliman research report): Milliman. Available online at: https://www.inseparable.us/AccessAcrossAmerica.pdf (Accessed March 31, 2025).

[ref24] Struijs J. N. Drewes H. W. Stein K. V. (2015). Beyond integrated care: challenges on the way towards population health management. Int. J. Integr. Care 15:e043. doi: 10.5334/ijic.2424, PMID: 27118960 PMC4843174

[ref25] Tepper M. C. Ward M. C. Aldis R. Lanca M. Wang P. S. Fulwiler C. E. (2022). Toward population health: using a learning behavioral health system and measurement-based care to improve access, care, outcomes, and disparities. Community Ment. Health J. 58, 1428–1436. doi: 10.1007/s10597-022-00957-3, PMID: 35352203 PMC8964387

[ref26] Van Beek K. Duchemin S. Gersh G. Pettigrew S. Silva P. Luskin B. (2008). Counseling and wellness services integrated with primary care: a delivery system that works. Perm. J. 12, 20–24. doi: 10.7812/TPP/08-038, PMID: 21339917 PMC3037136

[ref27] World Health Organization, (2022). Mental disorders. Available online at: https://www.who.int/news-room/fact-sheets/detail/mental-disorders (Accessed August 3, 2024).

